# Clopidogrel-induced neutropenia in an 80-year-old patient with chronic kidney disease who underwent percutaneous coronary intervention: a case report and literature review

**DOI:** 10.1186/s12872-022-02490-3

**Published:** 2022-02-11

**Authors:** Yannan Pan, Bing Liu, Junmeng Liu, Wei Zhuang, Qing He, Ming Lan

**Affiliations:** 1 School of Medicine, Peking University Health Science Center, Beijing, China; 2 Department of Cardiology, Beijing Hospital, National Center of Gerontology, Beijing, China; 3 Department of Medical Oncology, National Cancer Center/National Clinical Research Center for Cancer/Cancer Hospital, Chinese Academy of Medical Sciences and Peking Union Medical College, Beijing, China

**Keywords:** Clopidogrel, Neutropenia, CKD, PCI

## Abstract

**Background:**

Clopidogrel is a widely-used antiplatelet and acts as an adenosine diphosphate receptor inhibitor. Neutropenia is a rare but serious adverse effect of clopidogrel. It is unknown whether this adverse effect has any association with impaired kidney function.

**Case presentation:**

An 80-year-old male with chronic kidney disease was diagnosed with non-ST elevation myocardial infarction and underwent percutaneous coronary intervention. During hospitalization, the patient was diagnosed with contrast-induced nephropathy, treated symptomatically, and discharged with a back-to-baseline creatinine level. Two weeks later, the patient presented to the emergency department with fever and chills. Complete blood count showed leukopenia (0.84 × 10^3^/mm^3^) and severe neutropenia (0.13 × 10^3^/mm^3^). Blood cultures were positive for *Pseudomonas aeruginosa*. Clopidogrel was stopped immediately and switched into ticagrelor. Imipenem and granulocyte colony-stimulating factor were administered to the patient. The patient’s white blood cell and absolute neutrophil count were within the normal range after four days of treatment. The patient was discharged after a 10-day hospitalization, and his complete blood counts were normal during further follow-ups.

**Conclusions:**

Clopidogrel was the most likely primary cause of neutropenia in our case. The incidence of clopidogrel-induced neutropenia is low and the exact mechanism is not fully explained. We provide suggestions on the management of clopidogrel-associated neutropenia, and summarize all five cases of clopidogrel-induced neutropenia in patients with impaired kidney function.

## Background

Clopidogrel is often used as part of the dual antiplatelet therapy (DAPT) in patients with acute coronary syndrome or those undergoing percutaneous coronary intervention (PCI). Clopidogrel is an antiplatelet that inhibits the binding of adenosine diphosphate (ADP) to P2Y12 receptor [1]. Clopidogrel may cause potential hematological side effects, and neutropenia is a rare but serious adverse effect, with an observed incidence of 0.10% according to the CAPRIE trial [2]. Here, we report a case of clopidogrel-induced neutropenia in a patient with chronic kidney disease (CKD) who underwent PCI, and summarize all five cases of clopidogrel-induced neutropenia in patients with impaired kidney function.

## Case presentation

An 80-year-old male complained of intermittent chest pain for three weeks. During this period, he had been diagnosed with non-ST elevation myocardial infarction (NSTEMI). The patient had a past medical history of hypertension (treated with amlodipine and ramipril), type 2 diabetes mellitus (treated with glargine, aspart, and voglibose), and a two-year history of chronic kidney disease (stage 4). Due to the concern of deteriorating kidney function, he was given medical therapy before transferring to our hospital. He was treated with aspirin (100 mg per day), clopidogrel (75 mg per day), and isosorbide mononitrate (20 mg per day) for three weeks before admission.

**Fig. 1:**
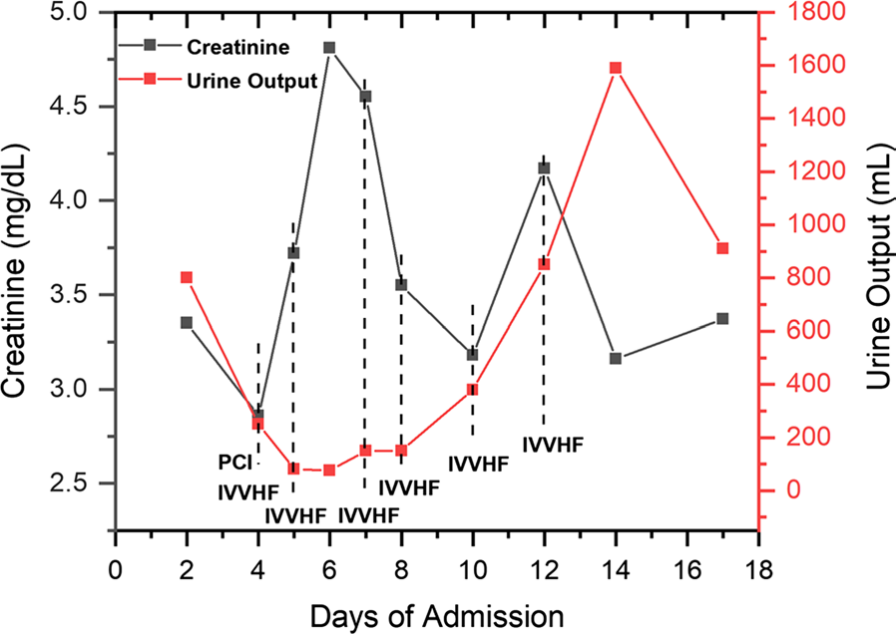
Periprocedural kidney function changes. *PCI* percutaneous coronary intervention, *IVVHF* intermittent venovenous hemofiltration

On admission, hematologic findings showed normal hemoglobin (12.3 g/dL), leukocyte count (7.85 × 10^3^/mm^3^), neutrophil count (5.13 × 10^3^/mm^3^), and platelet count (183 × 10^3^/mm^3^). His baseline creatinine level was 3.35 mg/dL and his estimated glomerular filtration rate (eGFR) was 16.42 mL/min/1.73 m^2^. Before the scheduled PCI, a loading dose of 300 mg clopidogrel and 300 mg aspirin were administered. During the procedure, two stents were placed in the left anterior descending artery, and two stents were placed in the right coronary artery. The patient received a maintenance dose of clopidogrel 75 mg per day and aspirin 100 mg per day after the procedure. Considering his low glomerular filtration rate (GFR), we administered intravenous isotonic normal saline 24 h pre-procedure and post-procedure, respectively, and the dose of contrast for PCI was 150 mL. Due to low urine output and elevated creatinine (from 3.35 to 4.81 mg/dL), the patient was given intermittent venovenous hemofiltration (IVVHF) for six times in all (Fig. 1). His hemoglobin dropped from 12.3 to 6.5 g/dL, which was most likely due to renal causes and blood loss during IVVHF, and he was given a three-unit red blood cell transfusion. In the meantime, his white blood cell and neutrophil count were in the normal range. On the 10th day after the PCI procedure, his urine output increased to 1590 mL and creatinine level decreased to 3.16 mg/dL (Fig. 1), and we stopped hemofiltration by then. He was then discharged with a back-to-baseline creatinine level of 3.37 mg/dL and a normal leukocyte count of 7.5 × 10^3^/mm^3^. His discharge medication lists included clopidogrel (75 mg per day), aspirin (100 mg per day), isosorbide mononitrate (30 mg per day), atorvastatin (20 mg per day), carvedilol (6.25 mg twice per day), furosemide (20 mg per day), and erythropoietin (10,000 IU three times per week subcutaneously).

On the 51st day of clopidogrel therapy, the patient went to the clinic for follow-up with no symptoms other than anorexia, and his complete blood count demonstrated a leukocyte count of 2.5 × 10^3^/mm^3^ and a neutrophil count of 1.0 × 10^3^/mm^3^. The patient was instructed to come to the clinic for close follow-ups of complete blood count. On the 55th day of clopidogrel treatment, he came to the emergency department complained of fever and chills with a temperature of 39.0 °C. He was somnolent but arousable. Vital signs showed that heart rate was 110, respiratory rate was 22, blood pressure was 99/58 mmHg, and O_2_ saturation was 100%. Complete blood count indicated leukopenia (0.84 × 10^3^/mm^3^) and severe neutropenia (0.13 × 10^3^/mm^3^). Since neutropenic fever is a potentially life-threatening condition, our priority was the initial assessment and prompt management of febrile neutropenia. Intravenous normal saline and supplemental oxygen were given. Two sets of blood cultures were drawn prior to intravenous empiric broad-spectrum antibiotics (imipenem/cilastatin). Subcutaneous granulocyte colony-stimulating factor (G-CSF) was administered due to the critical condition of this patient. Given the possibility of drug-induced neutropenia, clopidogrel was no longer used and was replaced by ticagrelor (90 mg twice per day). Further blood tests showed normal electrolytes and liver function tests, a creatinine level of 3.96 mg/dL, and elevated C-reactive protein of 18 ng/dL. We performed several laboratory tests and imaging studies to identify potential pathogen and source of infection. Two sets of blood cultures both came out positive for *Pseudomonas aeruginosa*, and antibacterial susceptibility tests proved the pathogen was susceptible to imipenem. Common viral studies were negative (CMV, EBV, influenza, HIV, hepatitis virus). As for the source of infection, the patient had no indwelling catheters, no skin breakdown, no abnormalities during oropharyngeal and perirectal exams, and complained no abdominal pain or diarrhea. Urine analysis was negative for white blood cells. Crackles were heard on auscultations, and chest X-ray and chest CT scans showed ground-glass opacities in the right lower lobe. Thus, the primary source of infection was most likely caused by pulmonary infection which later developed into sepsis.

We performed a thorough differential diagnosis of potential causes of neutropenia. Further laboratory evaluations were obtained, including vitamin B12 and folic acid levels, autoimmune antibodies, peripheral blood smears, and abdominal ultrasound (to rule out splenomegaly), and all of them showed no abnormalities. Common etiologies of neutropenia in the elderly includes infection, medication, nutritional deficiencies, hematologic malignancies, and autoimmune disorders [3]. Nutritional causes were ruled out since vitamin B12 and folic acid levels were within the normal range. Autoimmune disorders were unlikely due to negative autoimmune antibodies. As for hematologic causes, this patient’s rapid response to treatment and normal peripheral blood smears made hematologic malignancies less likely, and abdominal ultrasound showed a normal-sized spleen which ruled out hypersplenism. As for infections, viral serologies (CMV, EBV, influenza, HIV, hepatitis virus) were negative, but positive blood cultures demonstrated that *Pseudomonas* sepsis might be a potential cause of neutropenia. On the other hand, drug-induced neutropenia was another plausible explanation. Among all the medications he had used, clopidogrel, aspirin, and furosemide might lead to neutropenia. However, clopidogrel was the only medication started using for the first time within the past three months, which made other drugs unlikely to be the cause of neutropenia. Among these two plausible causes, the patient had no fever or symptoms of infection when neutropenia was first detected on the 51st day of clopidogrel treatment. Therefore, it was more likely that neutropenia occurred before sepsis rather than the opposite. Moreover, according to a monocentric cohort study, 34% of patients with drug-induced agranulocytosis primarily presented with septicemia or septic shock [4], and the percentage increased to 64% in elderly patients [5], indicating septicemia was not uncommon for patients with drug-induced neutropenia. Applying Naranjo ADR (adverse drug reaction) probability scale to this patient, we reached a score of 5, which indicated that clopidogrel was the probable cause [6]. That made clopidogrel the most likely primary culprit of neutropenia in this patient, and acute sepsis caused by inadequate bone marrow reserve might further exacerbate the progression of neutropenia.

**Fig. 2:**
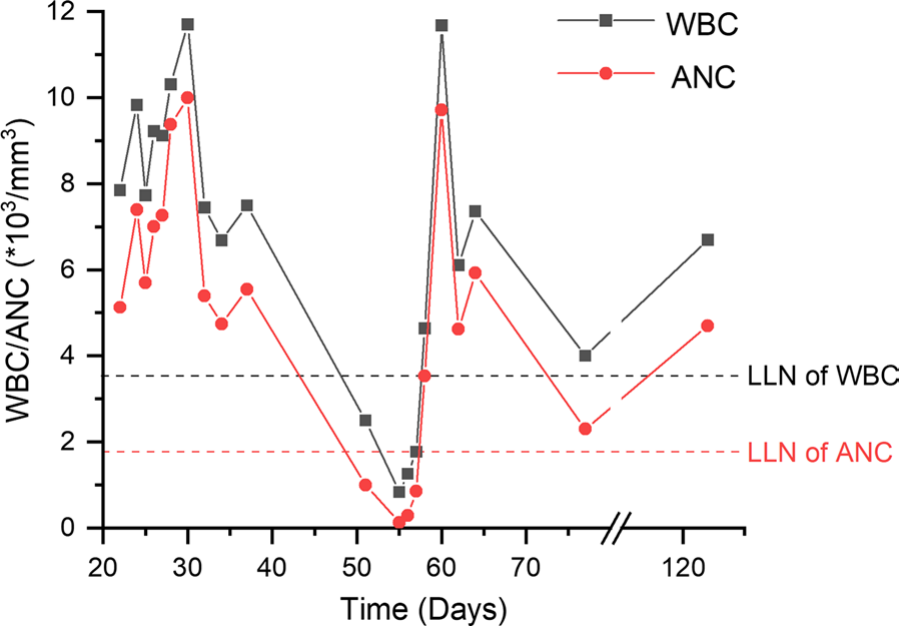
Time course of white blood cell and absolute neutrophil count after clopidogrel use. Clopidogrel was used from day 1 to day 55. Ticagrelor had been used since day 56. G-CSF was used from day 55 to day 58. *WBC* white blood cell, *ANC* absolute neutrophil count, *LLN* lower limits of normal

During hospitalization, we continued monitoring his vital signs, complete blood count, inflammatory markers, and cardiac biomarkers (Table 1). The change of white blood cell and absolute neutrophil count was shown in Fig. 2. After four days of G-CSF and imipenem/cilastatin treatment, he was clinically stable with a normal temperature and white blood cell count, and his absolute neutrophil count had recovered to 3.53 × 10^3^/mm^3^. Therefore, G-CSF and imipenem/cilastatin were discontinued, and antibiotics was switched into oral moxifloxacin, which was used for another four days. The patient’s clinical status, temperature, neutrophil count, and inflammatory markers were normal since then. He was discharged after 10 days of hospitalization, and was instructed to monitor complete blood count periodically. He came for follow-ups after using ticagrelor for 23 and 68 days, respectively, and his complete blood count was within the normal range. The patient reported no adverse effects associated with ticagrelor use.

**Table 1:** Serial analysis of complete blood count, temperature, inflammatory markers, and cardiac biomarkers

Days from clopidogrel use	Management	T (C)	WBC (× 10^3^/mm^3^)	ANC (× 10^3^/mm^3^)	Hb (g/dL)	PLT (× 10^3^/mm^3^)	CRP (mg/dL)	hsTnI (ng/mL)	BNP (pg/mL)
37		36.8	7.50	5.55	9.9	164	–	–	1030.11
51		Normal	2.5	1.0	13.4	137	–	–	–
55	Discontinued clopidogrel; started ticagrelor, G-CSF, and IV imipenem/cilastatin	39.0	0.84	0.13	9.0	137	18.4	0.14	1130.69
56		–	1.26	0.29	8.6	111	–	0.11	295.4
57		36.5	1.77	0.86	8.5	124	–	–	–
58	Discontinued G-CSF and imipenem/cilastatin; started oral moxifloxacin	36.5	4.64	3.53	9.0	133	8.4	–	–
60		36.3	11.68	9.71	9.6	189	3.4	0.06	759.22
62	Discontinued moxifloxacin	36.4	6.11	4.62	10.6	207	–	0.05	961.65
64	Discharged	36.2	7.36	5.93	10.4	233	–	–	–
77	Follow-up	Normal	4.0	2.3	12.2	165	–	–	–
122	Follow-up	Normal	6.7	4.7	13.5	137	–	–	–

*T* temperature, *WBC* white blood cell, *ANC* absolute neutrophil count, *Hb* hemoglobin, *PLT* platelet, *CRP* C-reactive protein, *hsTnI* high-sensitivity troponin I, *BNP* brain natriuretic peptide, *G-CSF* granulocyte colony-stimulating factor, *IV* intravenous

## Discussion and conclusions

Dual antiplatelet therapy (DAPT) includes aspirin plus clopidogrel. European Society of Cardiology guidelines suggest using dual antiplatelet therapy (DAPT) in patients diagnosed with acute coronary syndrome or patients undergoing percutaneous coronary intervention [7]. Clopidogrel may cause potential hematological adverse effects, and bleeding is the most common one. Other hematological side effects include neutropenia, thrombocytopenia, pancytopenia, thrombotic thrombocytopenic purpura, and hemolytic uremic syndrome [8]. According to the CAPRIE (Clopidogrel Versus Aspirin in Patients at Risk of Ischemic Events) [2] and CURE (Clopidogrel in Unstable Angina to Prevent Recurrent Events) studies [9], the incidence of neutropenia in patients treated with clopidogrel is low. In the CAPRIE trial, the observed incidence of neutropenia after using clopidogrel was 0.10%, and the incidence of severe neutropenia (< 450/mm^3^) was 0.05%. In the CURE studies, eight patients were reported with neutropenia in the clopidogrel group, which had 6259 patients in total, and the estimated incidence of neutropenia was 0.12%. Even though neutropenia is a rare adverse effect, the actual incidence could be underestimated until further investigation in large multi-center clinical trials [8].

The exact mechanism of clopidogrel-induced neutropenia was not fully explained. Previous bone marrow biopsies demonstrated that clopidogrel might cause neutropenia by inhibiting myeloid colony growth [10]. Other studies suggested two possible mechanisms including cumulative toxicity and idiosyncratic reaction [11]. There were more studies about the possible mechanisms of ticlopidine-induced neutropenia. Ticlopidine and clopidogrel are first and second generation thienopyridines, respectively. They have similar structures, but ticlopidine has a much higher neutropenia occurrence rate (2.1%) [12]. Thus, ticlopidine and clopidogrel might share similar mechanisms leading to neutropenia. Ono et al. [13] suggested ticlopidine exhibited an inhibitory effect on colony-forming unit in culture (CFU-C) directly and dose-dependently. This might be caused by a local increase in prostaglandin E1 produced by ticlopidine or result from an immunologic mechanism [14]. Maseneni et al. [15] demonstrated thienopyridines’ metabolites formed by myeloperoxidase led to the toxicity on neutrophil granulocytes. These metabolites caused reactive oxygen species accumulation and cell apoptosis.

According to Wu et al. [10], who summarized 12 cases from 2000 to 2014, the median age of clopidogrel-associated neutropenia patients was 65 years old. On average, neutropenia was detected after using clopidogrel for 22 days (ranging from 7 to 48 days), and the median neutrophil count at the time of onset was 479/mm^3^ (ranging from 0 to 1600/mm^3^). The recovery time was four days in those treated with G-CSF, while the recovery time was six days in those who was not treated with G-CSF. In our case, neutropenia was detected on the 51st day of clopidogrel therapy with a neutrophil count of 1000/mm^3^, and the nadir of neutrophil count was 130/mm^3^. We stopped clopidogrel therapy, switched to ticagrelor, and used empiric broad-spectrum antibiotics to treat sepsis. Several factors are correlated with poor prognosis in patients with drug-induced neutropenia, including age > 65 years old, preexisting comorbidities (especially renal insufficiency), septicemia, and absolute neutrophil count < 100/mm^3^ [16–18]. Due to multiple poor prognostic factors, we used G-CSF treatment in this patient. The patient’s neutrophil count recovered in four days. Even though neutropenia is an infrequent adverse effect of clopidogrel, clinicians should always keep its possibility in mind and check complete blood count during patient follow-ups. In summary of the reported cases, the presenting symptoms range widely from normal-being with no chief complaints to moderate tiredness to critical neutropenic fever. As for management, clopidogrel should be discontinued, and G-CSF could be used in patients with poor prognostic factors to speed the recovery time. Prevention of secondary infections and timely treatment of sepsis is a critical part of management as well. If neutropenic fever occurred, possible pathogen and source of infection should be identified, and temperature, complete blood count, and inflammatory markers should be followed up to decide treatment duration.

Reviews of literature suggest using several other antiplatelets when clopidogrel-associated neutropenia occurred, including prasugrel, cilostazol, and ticagrelor [19–21]. Prasugrel belongs to thienopyridine antiplatelets, and it has similar ring structures seen in clopidogrel and ticlopidine [19]. In TRITON-TIMI 38 trial, the incidence rate of neutropenia was less than 0.1% in the prasugrel group, whereas the incidence was 0.2% in the clopidogrel group [22]. On the other hand, ticagrelor is a nonthienopyridine antiplatelet and cilostazol is a phosphodiesterase-III selective inhibitor. Their structural differences from clopidogrel might support their uses in clopidogrel-associated neutropenia. In our case, we chose ticagrelor since there were no reports of neutropenia caused by ticagrelor. Our patient’s white blood cell and absolute neutrophil count have been in the normal range after switching into ticagrelor. However, there is no consensus on which medication is superior to choose when clopidogrel-induced neutropenia occurs, and more evidence is stilled needed.

What makes our patient special is that he had chronic kidney disease (stage 4) and suffered from contrast-induced nephropathy. After reviewing all the case reports of clopidogrel-associated neutropenia from Pubmed, we found a total of four cases of patients who had chronic kidney disease. The following table (Table 2) summarizes these four cases in addition to our patient. The average onset time of neutropenia in patients with chronic kidney disease was 36 days. Clopidogrel is metabolized in liver into its active metabolites and excreted by kidney [23]. Even though thienopyridines might cause neutropenia in a dose-dependent manner, whether this adverse effect has any association with impaired kidney function is still unknown. Careful clinical and hematologic monitoring is critical to patients treated with clopidogrel, especially during the first one to two months in those with chronic kidney disease [24]. Moreover, serum creatinine level > 1.36 mg/dL is one of the poor prognostic factors of drug-induced neutropenia [17], and patients with chronic kidney disease should be monitored closely and treated promptly.

**Table 2:** Summary of case reports of clopidogrel-induced neutropenia in chronic kidney disease patients

	Age (year)	Sex	Clopidogrel dose	eGFR (mL/min/1.73 m^2^)	Onset (days)	Recovery (days)	WBC at onset (/mm^3^)	ANC at onset (/mm^3^)
Chemnitz et al. [25]	35	F	75 mg	45	28	death	2000	N/A
Akcay et al. [26]	33	M	75 mg	8.10	21	5	1500	525
Suh et al. [24]	40	F	LD: 300 mg; 75 mg	N/A	48	5	2100	378
Wu et al. [10]	71	F	75 mg	N/A	32	4	250	0
Case	80	M	LD: 300 mg; 75 mg	16.42	51	4	2500	1000

*eGFR* estimated glomerular filtration rate, *WBC* white blood cell, *ANC* absolute neutrophil count, *N/A* not available, *LD* loading dose

In conclusion, clopidogrel was the most likely primary cause of neutropenia in this patient. The incidence of clopidogrel-induced neutropenia is low and the exact mechanism is not fully explained. It is unknown whether this adverse effect has association with impaired kidney function. We summarize all five cases of clopidogrel-induced neutropenia in patients with impaired kidney function. Careful clinical and hematologic monitoring to patients treated with clopidogrel should be suggested.

## Data Availability

The datasets supporting the conclusions of this article are included within the manuscript.

## Abbreviations

ADP: Adenosine diphosphate
ANC: Absolute neutrophil count
CKD: Chronic kidney disease
DAPT: Dual antiplatelet therapy
eGFR: Estimated glomerular filtration rate
G-CSF: Granulocyte colony-stimulating factor
IVVHF: Intermittent venovenous hemofiltration
NSTEMI: Non-ST elevation myocardial infarction
PCI: Percutaneous coronary intervention
WBC: White blood cell

## References

[1] Sharis PJ, Cannon CP, Loscalzo J (1998). The antiplatelet effects of ticlopidine and clopidogrel. Ann Intern Med.

[2] CAPRIE Steering Committee (1996). A randomised, blinded, trial of clopidogrel versus aspirin in patients at risk of ischaemic events (CAPRIE). Lancet.

[3] Boxer LA (2012). How to approach neutropenia. Hematol Am Soc Hematol Educ Progr.

[4] Andrès E, Maloisel F, Kurtz JE, Kaltenbach G, Alt M, Weber JC, Sibilia J, Schlienger JL, Blicklé JF, Brogard JM (2002). Modern management of non-chemotherapy drug-induced agranulocytosis: a monocentric cohort study of 90 cases and review of the literature. Eur J Intern Med.

[5] Andrès E, Noel E, Kurtz JE, Henoun Loukili N, Kaltenbach G, Maloisel F (2004). Life-threatening idiosyncratic drug-induced agranulocytosis in elderly patients. Drugs Aging.

[6] Naranjo CA, Busto U, Sellers EM, Sandor P, Ruiz I, Roberts EA, Janecek E, Domecq C, Greenblatt DJ (1981). A method for estimating the probability of adverse drug reactions. Clin Pharmacol Ther.

[7] Windecker S, Kolh P, Alfonso F, Collet JP, Cremer J, Falk V, Filippatos G, Hamm C, Head SJ, Jüni P (2014). 2014 ESC/EACTS Guidelines on myocardial revascularization: the Task Force on Myocardial Revascularization of the European Society of Cardiology (ESC) and the European Association for Cardio-Thoracic Surgery (EACTS)Developed with the special contribution of the European Association of Percutaneous Cardiovascular Interventions (EAPCI). Eur Heart J.

[8] Almsherqi ZA, McLachlan CS, Sharef SM (2007). Non-bleeding side effects of clopidogrel: have large multi-center clinical trials underestimated their incidence?. Int J Cardiol.

[9] Yusuf S, Zhao F, Mehta SR, Chrolavicius S, Tognoni G, Fox KK (2001). Effects of clopidogrel in addition to aspirin in patients with acute coronary syndromes without ST-segment elevation. N Engl J Med.

[10] Wu CW, Wu YJ, Wu CC (2016). Clopidogrel-associated neutropenia: case report and review of the literature. Am J Ther.

[11] Andres E, Perrin AE, Alt M, Goichot B, Schlienger JL (2001). Febrile pancytopenia associated with clopidogrel. Arch Intern Med.

[12] Quinn MJ, Fitzgerald DJ (1999). Ticlopidine and clopidogrel. Circulation.

[13] Ono K, Kurohara K, Yoshihara M, Shimamoto Y, Yamaguchi M (1991). Agranulocytosis caused by ticlopidine and its mechanism. Am J Hematol.

[14] Taher A, Ammash Z, Dabajah B, Nasrallah A, Mourad FH (2000). Ticlopidine-induced aplastic anemia and quick recovery with G-CSF: case report and literature review. Am J Hematol.

[15] Maseneni S, Donzelli M, Brecht K, Krähenbühl S (2013). Toxicity of thienopyridines on human neutrophil granulocytes and lymphocytes. Toxicology.

[16] Juliá A, Olona M, Bueno J, Revilla E, Rosselló J, Petit J, Morey M, Flores A, Font L, Maciá J (1991). Drug-induced agranulocytosis: prognostic factors in a series of 168 episodes. Br J Haematol.

[17] Andrès E, Maloisel F (2008). Idiosyncratic drug-induced agranulocytosis or acute neutropenia. Curr Opin Hematol.

[18] Maloisel F, Andrès E, Kaltenbach G, Noel E, Martin-Hunyadi C, Dufour P (2004). Prognostic factors of hematological recovery in life-threatening nonchemotherapy drug-induced agranulocytosis. A study of 91 patients from a single center. Presse Med.

[19] Shah R, Keough LA, Belalcazar-Portacio A, Ramanathan KB (2015). Ticagrelor as an alternative in clopidogrel-associated neutropenia. Platelets.

[20] Khangura S, Gordon WL (2011). Prasugrel as an alternative for clopidogrel-associated neutropenia. Can J Cardiol.

[21] Montalto M, Porto I, Gallo A, Camaioni C, Della Bona R, Grieco A, Crea F, Landolfi R (2011). Clopidogrel-induced neutropenia after coronary stenting: is cilostazol a good alternative?. Int J Vasc Med.

[22] Wiviott SD, Braunwald E, McCabe CH, Montalescot G, Ruzyllo W, Gottlieb S, Neumann FJ, Ardissino D, De Servi S, Murphy SA (2007). Prasugrel versus clopidogrel in patients with acute coronary syndromes. N Engl J Med.

[23] Balamuthusamy S, Arora R (2007). Hematologic adverse effects of clopidogrel. Am J Ther.

[24] Suh SY, Rha SW, Kim JW, Park CG, Seo HS, Oh DJ, Ro YM (2006). Neutropenia associated with clopidogrel use in a patient with chronic renal failure who underwent percutaneous coronary and peripheral intervention. Int J Cardiol.

[25] Chemnitz J, Söhngen D, Schulz A, Diehl V, Scheid C (2003). Fatal toxic bone marrow failure associated with clopidogrel. Eur J Haematol.

[26] Akcay A, Kanbay M, Agca E, Sezer S, Ozdemir FN (2004). Neutropenia due to clopidogrel in a patient with end-stage renal disease. Ann Pharmacother.

